# Hydroxychloroquine potentiates carfilzomib toxicity towards myeloma cells

**DOI:** 10.18632/oncotarget.12226

**Published:** 2016-09-23

**Authors:** Katarzyna Baranowska, Kristine Misund, Kristian K. Starheim, Toril Holien, Ida Johansson, Sagar Darvekar, Glenn Buene, Anders Waage, Geir Bjørkøy, Anders Sundan

**Affiliations:** ^1^ Department of Cancer Research and Molecular Medicine, Faculty of Medicine, Norwegian University of Science and Technology, Trondheim, Norway; ^2^ CEMIR–Center of Molecular Inflammation Research, Faculty of Medicine, Norwegian University of Science and Technology, Trondheim, Norway; ^3^ Department of Laboratory Medicine, Children's and Women's Health, Faculty of Medicine, Norwegian University of Science and Technology, Trondheim, Norway; ^4^ Department of Hematology, St. Olav's University Hospital, Trondheim, Norway; ^5^ Department of Medical Laboratory Technology, Faculty of Technology, Norwegian University of Science and Technology, Trondheim, Norway

**Keywords:** myeloma, proteasome, carfilzomib, bortezomib, resistance

## Abstract

Cells degrade proteins either by proteasomes that clinically are targeted by for example bortezomib or carfilzomib, or by formation of autophagosomes and lysosomal degradation that can be inhibited by hydroxychloroquine (HCQ). Multiple myeloma is unique among cancers because proteasomal inhibition has good clinical effects. However, some multiple myeloma patients display intrinsic resistance to the treatment and most patients acquire resistance over time. We hypothesized that simultaneous targeting both arms of protein degradation could be a way to improve treatment of multiple myeloma. Here we tested the combined effects of the lysosomal inhibitor HCQ and clinically relevant proteasome inhibitors on myeloma cell lines and primary cells. Carfilzomib and bortezomib both induced immunoglobulin-containing aggregates in myeloma cells. HCQ significantly potentiated the effect of carfilzomib in both cell lines and in primary myeloma cells. In contrast, HCQ had little or no effects on the toxicity of bortezomib. Furthermore, cells adapted to tolerate high levels of carfilzomib could be re-sensitized to the drug by co-treatment with HCQ. Thus, we show that inhibition of lysosomal degradation can overcome carfilzomib resistance, suggesting that the role of autophagy in myeloma cells is dependent on type of proteasome inhibitor. In conclusion, attempts should be made to combine HCQ with carfilzomib in the treatment of multiple myeloma.

## INTRODUCTION

Multiple myeloma is one of a few cancers where inhibition of proteasome activity is an efficient treatment strategy. Thus, the introduction of bortezomib (Velcade) more than 10 years ago has led to substantial prolongation of survival of myeloma patients [1]. However, not all patients respond to bortezomib, and over time most patients become resistant to the treatment. A number of second-generation proteasome inhibitors such as the recently approved drug carfilzomib have been developed [2, 3]. Intriguingly, it is not necessarily the same patients that benefit from bortezomib and carfilzomib treatment, indicating that the drugs may have different and unknown determinants of sensitivity and resistance [4].

Multiple myeloma is an incurable malignancy of terminally differentiated B cells and is characterized by long-lived, slowly proliferating malignant plasma cells in the bone marrow. Overproduction and misfolding of monoclonal immunoglobulin results in formation of intracellular protein aggregates, and the presence of unfolded proteins and protein aggregates are indeed hallmarks of both normal and malignant plasma cells [5]. For plasma cells to survive, the unfolded protein response is constitutively activated [6, 7]. Thus, the particular sensitivity of myeloma cells towards inhibition of proteasomes may be due to the high levels of monoclonal immunoglobulin production and the accompanying stress [6]. Extensive attempts to target various parts of the protein degradation machinery for treatment of multiple myeloma have been explored [7, 8]. In principle, eukaryotic cells have two distinct but interconnected mechanisms for protein degradation and removal of misfolded proteins or protein aggregates [9, 10]. One is the ubiquitin-proteasome-system where proteins are poly-ubiquitinated for subsequent degradation by proteasomes. This pathway is targeted by drugs such as bortezomib and carfilzomib. The second one is sequestration of protein aggregates into autophagosomes and subsequent lysosomal degradation, a process known as macro-autophagy (hereafter referred to as autophagy). Cargo targeted for degradation by autophagy may also be labeled by ubiquitin [9]. These degradation pathways can compensate for each other, but the underlying mechanisms are unclear [10]. Treatment of plasma cells with proteasomal inhibitors lead to accumulation of immunoglobulin-containing aggregates, and autophagy has been indicated to be important in removal of such aggregates [5].

There have been several attempts of targeting autophagy/lysosomal protein degradation for disease treatment [11, 12]. A number of phase I clinical trials have tested the lysosomal inhibitor hydroxychloroquine (HCQ) [13], either used alone or combined with various chemotherapeutic drugs, in different types of cancers. HCQ is an attractive drug in this respect as it is already approved for clinical use in treatment of malaria. HCQ is known to increase lysosomal pH, thereby inhibiting protein hydrolysis and fusion of lysosomes with autophagosomes. Results of a phase I clinical trial combining bortezomib and HCQ in myeloma treatment has recently been reported [14].

Here, we investigate if autophagy plays a role for myeloma cell survival under conditions of proteasomal inhibition. Treatment with both bortezomib and carfilzomib resulted in accumulation of approximately similar levels of immunoglobulin-containing aggregates in myeloma cells. Interestingly, inhibition of autophagy and lysosomal protein degradation with HCQ or Bafilomycin A1 (BafA1) potentiated carfilzomib toxicity towards myeloma cells, whereas little or no additional effect were obtained by adding HCQ to cells treated with bortezomib. Cells selected to tolerate high amounts of carfilzomib were re-sensitized to carfilzomib when treated in the presence of HCQ. Furthermore, cells selected for carfilzomib resistance expressed significantly elevated levels of the autophagy receptor SQSTM1 (p62), and conversely, overexpression of SQSTM1 increased tolerance to carfilzomib-induced cytotoxicity. Our findings suggest that autophagy plays a role for cell survival in carfilzomib-treated cells and provide a rationale for clinical trials with treatment combining carfilzomib and HCQ in both carfilzomib-naïve and -resistant myeloma patients.

## RESULTS

### Myeloma cells contain intracellular aggregates and aggregate formation is strongly augmented by proteasome inhibitors

**Figure 1:**
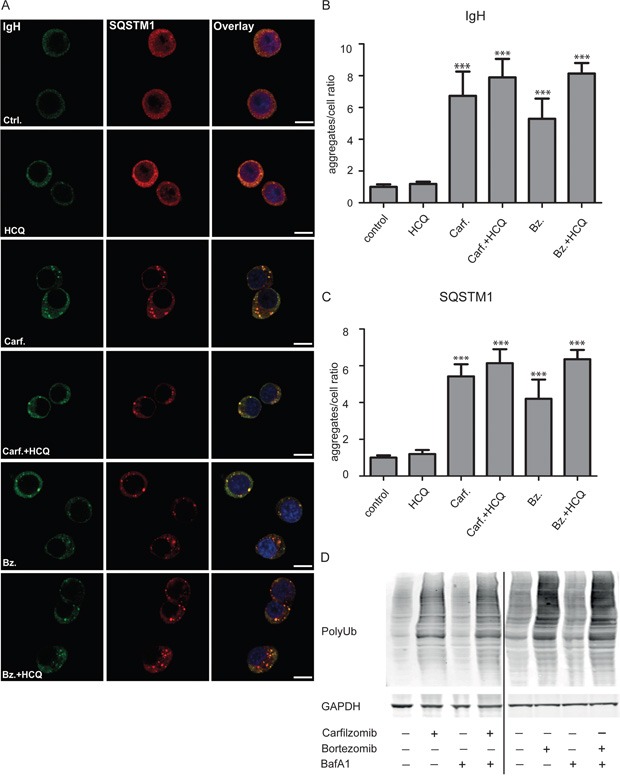
Proteasome inhibitors lead to major increase in IgH and SQSTM1–containing aggregates in myeloma cells **A.** ANBL-6 cells were incubated with and without HCQ (20 μM), carfilzomib (Carf. 15 nM), bortezomib (Bz, 7 nM) as indicated for 13 hrs. Cells were fixed and stained with primary antibodies towards IgH and SQSTM1. DNA was visualized using Hoechst 33342 staining. Scale bar is 10 μm. The staining pattern was homogenous for the different conditions and the images are representative for more than 300 cells manually inspected in the confocal microscope. **B** and **C.** Aggregates in cells shown in (A) were quantified by counting numbers of aggregates stained with anti IgH (B) and SQSTM1 (C) using automated fluorescence image capture and analysis by the ScanR microscope. Results are expressed as aggregate/cell as the mean +- SD (Standard Deviation) of 5 independent experiments. A two-way between groups analysis of variance (ANOVA) was used to compare aggregate amounts among the groups. The asterisks indicate p<0.001 in the comparisons of either untreated or HCQ-treated groups with the other groups. **D.** Both carfilzomib and bortezomib induce accumulation of ubiquitinated proteins. ANBL6 (4 left-hand lanes) and INA6 (right-hand lanes) cell lines were treated with 4 nM carfilzomib, 4 nM bortezomib for 24 hours, and/or 90 nM bafilomycin A1 (BafA1) for 18 hours, as indicated, before cell lysis in 8 M Urea, electrophoresis, and analysis by immunoblotting.

When untreated ANBL-6 myeloma cells were stained with anti-immunoglobulin heavy chain (IgH), distinct spots in the cell cytosol could be observed (Figure 1A and Supplementary Figure S1). This observation of cytosolic immunoglobulin-containing aggregates is in line with earlier reported observations in antibody-producing plasma cells [5]. Spots were also positive for SQSTM1 (p62) (Figure 1A) and conjugated ubiquitin (not shown). SQSTM1 is an autophagy-receptor that collects misfolded proteins into aggregates and links the aggregates to the forming autophagic membrane [15, 16]. Moreover, the numbers of IgH- and SQSTM1-containing aggregates were similar (Figure 1B, 1C), and most of the aggregates were double positive for IgH and SQSTM1 (Figure 1A).

Treatment of the cells with either carfilzomib or bortezomib significantly increased the number of aggregates (Figure 1A-1C) as well as the levels of ubiquitinated proteins in the cells (Figure 1D). In contrast, HCQ (Figure 1B, 1C) or BafA1 (data not shown) did not affect the number of aggregates or protein ubiquitin levels in the cells, nor did HCQ significantly affect the level of aggregates seen in the presence of either carfilzomib or bortezomib. Control experiments showed that HCQ treatment under similar conditions led to accumulation of LC3B-II in the cells (Supplementary Figure S2). Taken together, these observations suggest that autophagy and lysosomal degradation plays only a minor role in preventing accumulation of aggregates both in untreated cells and when proteasomes are inhibited, and that proteasomes play a decisive role in preventing accumulation of IgH/SQSTM1-containing aggregates in plasma cells.

In experiments shown in Figure 1, cells were treated with concentrations of carfilzomib or bortezomib that were equally toxic to the cells when cell viability was measured the next day (data not shown). The number of aggregates observed before cell toxicity could be observed, was not significantly different in cells treated with carfilzomib compared to cells treated with bortezomib (Figure 1B, 1C). Thus, with respect to the degree of accumulation of aggregates upon treatment with proteasomal inhibitors, no major differences between carfilzomib and bortezomib were detected.

The ANBL-6 cells are known to secrete only immunoglobulin light chain [17]. However, it is clear from these observations that they also make immunoglobulin heavy chains that accumulate in SQSTM1-containing aggregates even in untreated cells (Figure 1). Thus, in these cells, IgH chains are made but presumably not folded properly to generate intact, soluble immunoglobulin, but rather continuously degraded by proteasomes and collected into aggregates containing SQSTM1 upon proteasomal inhibition.

### HCQ potentiates the cytotoxic effect of carfilzomib on several multiple myeloma cell lines

**Figure 2:**
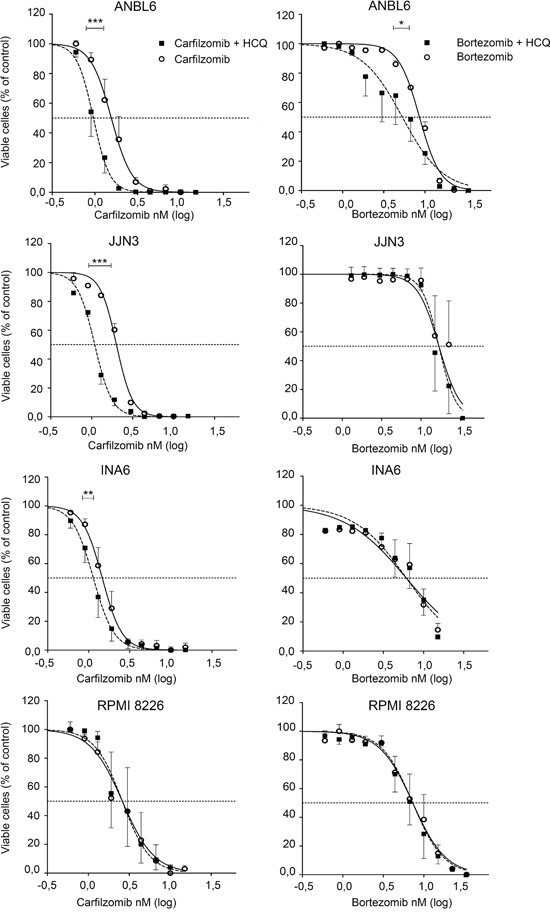
HCQ potentiates carfilzomib, but not bortezomib induced cell death in myeloma cell lines ANBL-6, JJN-3, INA-6 and RPMI-8226 cell lines were incubated with carfilzomib or bortezomib with or without HCQ (10 μM) for 3 days before cell viability was determined after staining of cell with YO-PRO-1 dye and estimated as described in Materials and Methods. Results are shown as the mean +-SEM (Standard Error of the Mean) of 3 independent experiments. Curves were fitted using non-linear regression. All IC50 were calculated with the use of non-linear regression analysis. Extra sum-of-squares F test was used to test whether IC50 values differed between groups. P values of the statistical comparison between IC50 values are indicated (*, p<0.05; **, p<0.01; *** p<0.001).

We hypothesized that proteasome inhibition produces toxic protein aggregates, and that autophagosomal/lysosomal breakdown of these ameliorates the cytotoxicity. We tested if concomitant inhibition of autophagy could potentiate cell toxicity obtained with proteasomal inhibitors. As shown in Figure 2, and Table 1, sub-lethal concentrations of HCQ significantly potentiated carfilzomib-induced apoptosis in 3 out of 4 examined myeloma cell lines. Likewise, Bafilomycin A1 potentiated carfilzomib toxicity (Supplementary Figure S1). Surprisingly, HCQ had little effects on bortezomib-induced cell death (Figure 2 and Table 1). At doses above 15-20 μM, HCQ alone induced some toxicity in these cell lines. However, combining higher doses of HCQ with bortezomib resulted only in additive effects and not in potentiation of bortezomib toxicity (data not shown). Taken together, the results indicate that the effect of carfilzomib in these cell lines can be dependent on autophagic-lysosomal protein degradation, whereas the effect of bortezomib appears to be relatively independent on lysosomal activity.

**Table 1:** Carfilzomib- and bortezomib toxicity the absence or presence of 10 μM HCQ

IC50 (nM)			
	CARFILZOMIB	CARF + HCQ	P
INA-6	1,46	1,14	0,0016
ANBL-6	1,55	0,96	< 0,0001
JJN-3	2,00	1,09	< 0,0001
RPMI-8226	2,41	2,54	0,8913
	BORTEZOMIB	BORT + HCQ	
INA-6	6,04	6,25	0,9689
ANBL-6	8,57	5,31	0,0125
JJN-3	17,0	15,3	0,3153
RPMI-8226	7,26	6,69	0,3804

Because HCQ has been reported to not only inhibit lysosomes, but also to allosterically affect proteasome activity, we tested the effect of HCQ on isolated proteasomes either alone or in the presence of carfilzomib or bortezomib [3]. In line with earlier reported results, we found that HCQ affected proteosomal protein degradation alone but only at concentrations above 80 μM (data not shown). Importantly, low doses of HCQ did not potentiate the inhibition of isolated proteasomes by carfilzomib, neither did low doses of HCQ affect bortezomib activity on proteasomes (Supplementary Figure S1C and data not shown). Thus, the potentiating effect of HCQ on carfilzomib activity is not due to potentiation of the carfilzomib interaction with proteasomes.

### Acquired carfilzomib tolerance in myeloma cell lines can be reversed by treatment with HCQ

**Figure 3:**
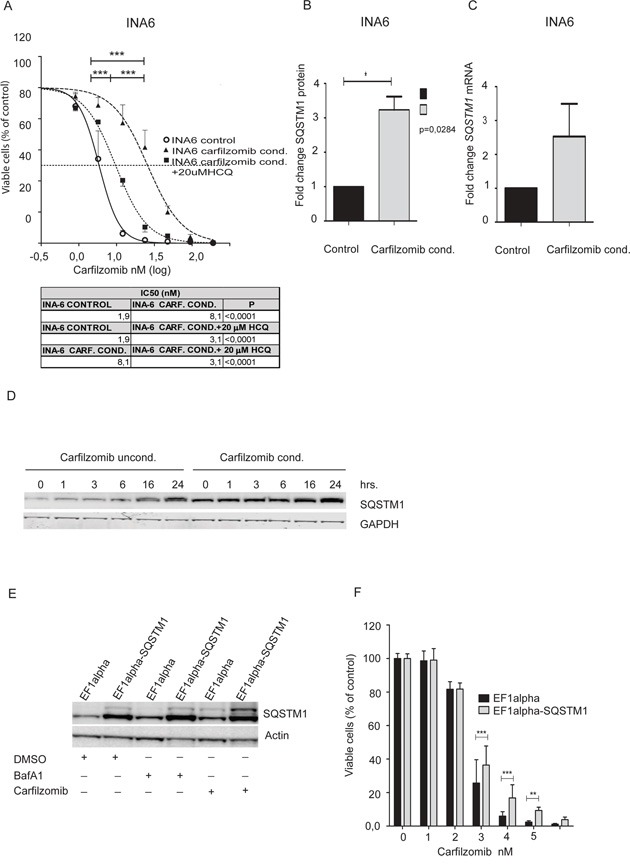
Carfilzomib conditioned INA6 cells show increased tolerance to carfilzomib that is partially reversed by HCQ **A.** INA-6 control cells and INA-6 carfilzomib- tolerant cells were incubated with carfilzomib for 16 hours with or without 20 μM HCQ as indicated. The ScanR microscope was used to measure cell death and the statistical analysis was performed as above. Shown are aggregate results from 3 independent experiments. **B.** Carfilzomib tolerant INA-6 cells and control cells were lysed, separated by gel electrophoresis and SQSTM1/GAPDH ratios were estimated by scanning the immunoblots. Shown are aggregated normalized mean +- SD (Standard Deviation) of 3 independent experiments. The asterisk indicates statistical significance (p<0.05, Student t-test). **C.** RNA was collected from carfilzomib-tolerant INA-6 cells and control cells and analyzed for *SQSTM1* mRNA levels. Results are calculated from 3 independent experiments; normalized and presented as fold change of relative *SQSTM1* mRNA levels between non-conditioned INA-6 cells and carfilzomib conditioned INA-6 cell line (mean + SD). **D.** Carfilzomib-conditioned INA6 cells and control cells were treated with 90 nM BafA1 for the indicated time points. Cells were lysed and SQSTM1-levels were determined by immunoblotting. **E.** INA-6 cells stably overexpressing SQSTM1 (EF1 alpha-SQSTM1) and control cells (EF1 alpha) were treated with carfilzomib (15 nM), BafA1 (90 nM) as indicated. After 8 hours cells were lysed and SQSTM1 levels were determined by immunoblotting. Actin was used as loading control. Results displayed are representative of 3 independent experiments. **F.** SQSTM1-overexpressing and control INA-6 cells were treated with indicated doses of carfilzomib overnight before evaluation of cell viability using the CellTiter-Glo® Luminescent Cell Viability Assay. Results are shown as the mean +-SD of 3 independent experiments. The asterisks indicate statistically significant differences (a two-way between groups analysis of variance (ANOVA)), *** indicates p<0.001, ** indicates p<0.01.

To further investigate mechanisms behind cell resistance towards carfilzomib we generated INA-6 cells tolerating high doses of carfilzomib (as described in the methods-section). These cells have been maintained without carfilzomib for >9 months, and they still tolerate significantly higher doses of carfilzomib than the control cells as shown in Figure 3A. Interestingly, combining carfilzomib with HCQ reversed most of the tolerance towards carfilzomib. Furthermore, as shown in Figure 3B and 3D, INA-6 cells tolerating higher levels of carfilzomib expressed significantly higher basal levels of SQSTM1 protein compared to control cells. Higher SQSTM1 protein levels in the carfilzomib-conditioned cells were also reflected in a trend towards higher SQSTM1 mRNA levels (Figure 3C). Both the sensitive and tolerant cell lines accumulate SQSTM1 protein levels at approximately the same rate over time upon lysosomal inhibition (Figure 3D), indicating that increased SQSTM1 levels in the carfilzomib-conditioned cells were not due to lower SQSTM1 degradation. Taken together, these results suggest that myeloma cells can acquire tolerability to carfilzomib by increasing basal levels of SQSTM1.

To further see if increased expression of SQSTM1 in INA-6 cells rendered them more tolerant to carfilzomib, we made cells stably overexpressing SQSTM1 from the EF1alpha promoter. As shown in Figure 3E, the cells had approximately four-fold higher levels of SQSTM1 protein, and as in control cells, the SQSTM1 protein turnover was dependent on autophagy and lysosomal degradation. Interestingly, the cells overexpressing SQSTM1 tolerated significantly higher amounts of carfilzomib (Figure 3F). Taken together, the results suggest that upregulation of basal levels of SQSTM1 protein could be sufficient to mediate resistance towards carfilzomib treatment. Interestingly, no difference in the turnover of LC3B-II was observed when comparing the carfilzomib sensitive and tolerant cells (data not shown). Thus, the ability of SQSTM1 to homo-polymerize and sequester misfolded proteins may affect cell survival without a change in the turnover of LC3B-II via autophagy.

### HCQ potentiate carfilzomib-induced apoptosis in primary myeloma cells

**Figure 4:**
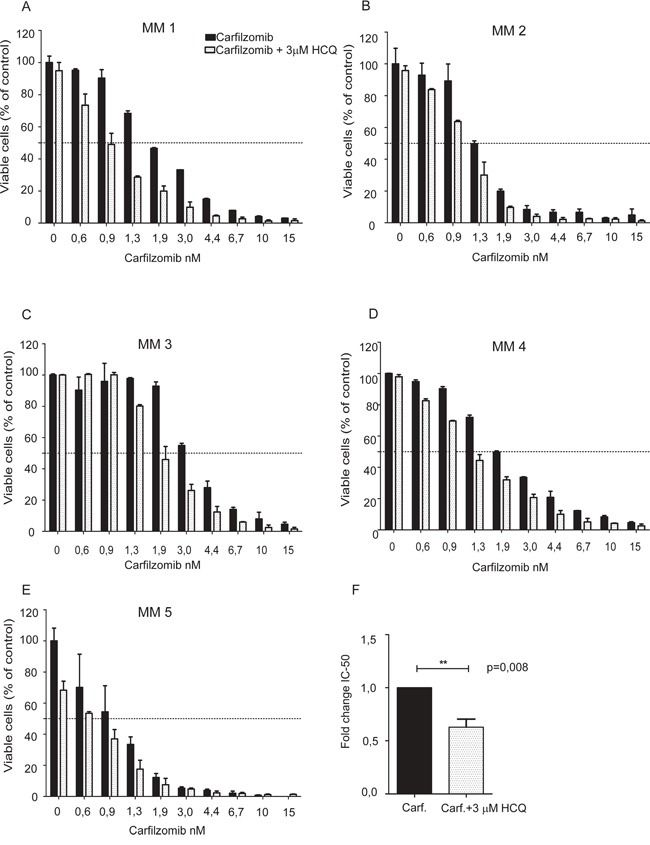
HCQ potentiates the carfilzomib-induced apoptosis in primary myeloma cells **A-E.** Isolated CD138^+^ plasma cells from 5 multiple myeloma patients were seeded in 96-well plates and incubated for 3 days with carfilzomib in the presence or absence of 3 μM HCQ. Plasma cell apoptosis was measured using automated fluorescence image capture and analysis by the ScanR microscope as described previously. Error bars indicate the standard deviation (SD) of duplicate measurements. **F.** IC50 values for the 5 primary myeloma samples was calculated using non-linear regression both for cells treated with carfilzomib alone or in combination with HCQ. After normalization, the extra sum-of-squares F test was used to test whether IC50 values differed between cells treated with or without HCQ. (Asterisks indicate p<0.05, Student t-test).

To investigate whether the ability of HCQ to potentiate the effects of carfilzomib is not only confined to relatively rapidly proliferating cell lines, we also tested the effects of combining carfilzomib and HCQ-treatment on 5 isolates of CD138^+^ primary myeloma cells, as previously described [18]. In all patient isolates tested there was a tendency towards increased cell death in cells treated with the combination of drugs compared to cells treated with carfilzomib alone. As expected, in primary myeloma cells isolated from different patients, the degree of the potentiating effect of HCQ on carfilzomib-induced cell death varied (Figure 4A-4E). However, when the 5 isolates were grouped, the HCQ induced a highly significant reduction of carfilzomib IC50 (Figure 4F).

## DISCUSSION

We here show that HCQ potentiate the cytotoxic effect of carfilzomib on myeloma cells. Furthermore, treatment with HCQ could partly reverse carfilzomib resistance in an *in vitro* carfilzomib resistance model. Thus, the combined treatment of carfilzomib and HCQ should be tested in the treatment of multiple myeloma patients whereas our results suggest that less might be gained by combining bortezomib with HCQ.

HCQ is a relatively inexpensive drug that inhibits autophagosomal fusion with lysosomes [14]. HCQ, being a weak base, acts by increasing lysosomal pH, and will thus inhibit not only degradation of cargo delivered to lysosomes by autophagosomes, but also general protein degradation in lysosomes. The drug has a well-known toxicity profile due to its application in treatment of malaria. However, when combined with an irreversible proteasome inhibitor such as carfilzomib, new types of toxicity may occur, and the combined use of these drugs warrants toxicity studies in myeloma patients.

We demonstrate a close interaction of the two arms of the protein degradation machinery in myeloma cells, indicating that autophagic and lysosomal degradation is critically important when proteasomes are inhibited with carfilzomib. The differential effects of inhibition of autophagy that we find in cells treated with carfilzomib *versus* bortezomib is indeed puzzling. Bortezomib and carfilzomib did not differ in their ability to induce accumulation of IgH- and SQSTM1-containing aggregates, suggesting that there is no straightforward relationship between removal of such presumably toxic aggregates and autophagy. While bortezomib interacts with proteasomes in a reversible manner, carfilzomib is an irreversible inhibitor. Thus, there may be a requirement for removal of irreversibly inactivated proteasomal proteins in cells treated with carfilzomib. Autophagy is important for degradation of intracellular organelles such as mitochondria, endoplasmatic reticulum, ribosomes, and peroxisomes [11]. The route of degradation of proteasomes in plasma cells has not been identified, but the lysosomal pathway was suggested to accomplish this task in other cell types [19]. Furthermore, proteomic analysis of the MCF-7 breast cancer cell line recently indicated the presence of proteasomal proteins within autophagosomes [20]. A likely route for degradation of proteasomes is therefore by autophagy and lysosomal degradation. Alternatively, dysfunctional proteasomes are dismounted and degraded by functional proteasomes. This way of proteosomal turnover could happen even in the presence of a reversible proteosomal inhibitor but be precluded by irreversible inhibitors. Accumulation of irreversibly inhibited proteasomes could represent a cellular stress by itself that synergizes with accumulation of damaged cellular components that should be turned over by autophagy. Taken together, the results presented here points to an important distinction between the effects of proteasome inhibitors in myeloma cells. However, further experiments must be designed to uncover the mechanisms for the apparent differential effects of proteosomal inhibitors.

The results presented here showing permanently elevated levels of SQSTM1 in cells made tolerant to carfilzomib suggest that the inherent plasma cell SQSTM1 protein levels may affect the efficacy of carfilzomib treatment. SQSTM1 is known as a protein induced by cellular stress (e.g. the presence of protein aggregates) driven by the transcription factor NRF2 [21, 22]. SQSTM1 collects misfolded proteins into aggregates on the forming autophagosome membrane. SQSTM1 protein levels in myeloma cells could possibly represent a marker of carfilzomib sensitivity. In malignant plasma cells, SQSTM1 levels could be affected by several factors such as nutrient starvation or the degree of protein aggregation in the cells. The degree of protein aggregation will likely vary between plasma cell clones dependent on the folding and solubility properties of the monoclonal heavy and light chains as well as the ratio of protein synthesis of heavy and light chains. However, it should be noted that resistance to carfilzomib likely may arise also by several other mechanisms than by increased levels of SQSTM1 as shown here.

The lack of synergy by combining bortezomib and HCQ treatment *in vitro* as described here is in line with earlier reported results that actually reported antagonistic effects of HCQ and bortezomib in some myeloma cell lines [23]. Based on the *in vitro* findings here it is unlikely that combined treatment with bortezomib and HCQ will be of advantage. During our experiments, a phase 1 clinical trial testing the effect of combining HCQ and bortezomib in multiple myeloma patients was published [14]. The combined treatment was tolerated relatively well by the patients, but presence of HCQ did not increase the effects of bortezomib in that study. Also there is one ongoing trial in refractory myeloma patients combining bortezomib and chloroquine [24]. Taken together, the results obtained suggest that HCQ should be combined with carfilzomib, and not with the reversible proteasome inhibitor bortezomib, in attempts to improve treatment of myeloma patients.

## MATERIALS AND METHODS

Stock solutions of carfilzomib (Active Biochemicals CO, Wan Chai, Hong Kong) and bortezomib (Selleck Chemicals, Munich, Germany) were prepared in dimethyl sulfoxide (Sigma–Aldrich, Schnelldorf, Germany). Hydroxycloroquine (HCQ) and Bafilomycin A1 (BafA1), both from Sigma-Aldrich, were dissolved in H_2_O, and ethanol, respectively.

### Myeloma cell lines and primary myeloma cells

Multiple myeloma cell lines ANBL-6, INA-6 and JJN3 were kind gifts from Dr. Diane Jelinek (Mayo Clinic, Rochester, MN), Dr. Martin Gramatzki (University of Erlangen-Nuremberg, Erlangen, Germany), and Dr. Jennifer Ball (University of Birmingham, UK), respectively. RPMI-8226 cells were obtained from ATCC (Rockville, MD, USA). ANBL-6 and INA-6 cells were grown in 10 % heat inactivated fetal calf serum (FCS) in RPMI-1640 (RPMI) medium containing 1 ng/ml Interleukin (IL)-6. JJN3 and RPMI-8226 cells were maintained in RPMI medium containing 10 and 20 % FCS, respectively. Cells were cultured at 37 °C in a humidified atmosphere containing 5 % CO_2_.

Bone marrow aspirates were obtained from Norwegian Myeloma Biobank (St. Olav's University Hospital HR, Trondheim, Norway). Myeloma cells were isolated from bone marrow aspirates from 5 patients using RoboSep automated cell separator and Human CD138 Positive Selection Kit (StemCell Technologies, Grenoble, France). Three of the 5 patients were treatment naïve whereas two of the samples were taken at relapse from a patients previously treated. A table describing the five patients is included in Supplementary materials (Supplementary Table S1). The purity of plasma cell isolates as estimated by counting plasma cells on cytospins was >95 %. The study was approved by the Regional Ethics Committee (REK2012/1509), and all patients had given informed consent.

### Carfilzomib tolerant INA-6 cells and SQSTM1 overexpressing cells

INA-6 cells were cultivated in media with an initial dose of 3 nM carfilzomib. Cells were split twice every week, and the carfilzomib dose was gradually increased with about 1 nM/month to a final dose of 10 nM after 6 months. Cells were then maintained in 10 nM carfilzomib for 3 months, and then maintained in carfilzomib-free medium for 4 weeks before being subjected to experiments. Control cells were cultivated similarly but without carfilzomib. The plasmid pENTR-SQSTM1 (Kan^R^) was a kind gift from Prof. Terje Johansen's laboratory (Molecular Cancer Research Group, The Arctic University of Norway). The lentiviral vector pLVX-EF1 alpha-IRES-ZsGreen 1 was purchased from Clontech (Catalog no. 631982). The gateway cassette was introduced at the XbaI site in the lentiviral vector. The sequence for the cassette is available on request. By simple gateway LR recombination reaction the SQSTM1 gene was cloned into the lentiviral vector. The empty pLVX-EF1 alpha-IRES-ZsGreen 1 vector or the pLVX-EF1 alpha-SQSTM1-IRES-ZsGreen 1 vector were packaged into viral particles in HEK293T cells, using supporting plasmids psPAX2, and pMD2.G. Virus particles in supernatants from HEK293T cells were transduced into the target cells INA6 applying polybrene. The cells were sorted based on ZsGreen 1 fluorescence. SQSTM1 and GAPDH mRNA levels were estimated by qRT-PCR after isolation of total RNA (RNAEasy kit, Qiagene) applying commercial Taqman kits (ThermoFisher Scientific, cat. no. 4331182).

### Confocal microscopy and aggresome quantification using automated fluorescence imaging

ANBL-6 cells were seeded in poly-L-lysine coated 96 well glass bottom plates (In Vitro Scientific, Sunnyvale, CA, USA) and left untreated or incubated with HCQ (10 μM), carfilzomib (15 nM), bortezomib (7 nM), or combinations as indicated for 13 h before the cells were fixed in 4 % paraformaldehyde. Cells were stained overnight in 2% BSA in PBS with primary antibodies towards IgH (Goat, dilution 1:200, Jackson Immuno-research, cat.no. 120172) and SQSTM1 (Rabbit, dilution: 1:300, MBL International, cat. no. PM045). Secondary antibodies were donkey anti-goat Alexa Fluor 555 (Dilution; 1:1000 in 2% BSA in PBS, Life Technologies, cat.no 1697092) and chicken anti-rabbit Alexa Flour 647 (Dilution; 1:1000 in 2% BSA in PBS, Life Technologies cat. no 1719643). For control cells, a cocktail of the two secondary antibodies was used to verify specificity of the antibody staining. A figure demonstrating antibody staining specificity is included in Supplementary materials (Supplementary Figure 1A).

Numbers of aggregates in cells were estimated applying a ScanR (Olympus, Hamburg, Germany) modular epifluorescence microscope. For each well, 72 different fields of view were analyzed, corresponding to approximately 4000 cells counted/well. The images were analysed by the ScanR Analysis software (Olympus) estimating the number of cells (based on the nuclear-stain) as well as the number and intensity of SQSTM1 and IgH dots within cells. In parallel, identically stained cells were imaged by confocal microscopy. Confocal images were captured using a Leica SP8 inverted microscope (Leica Microsystems, Mannheim, Germany) equipped with a HC plan-apochromat 63×/1.4 CS2 oil-immersion objective.

### Cell viability assays

Cell death in cell lines and primary myeloma cell isolates was measured essentially as earlier described [18]. Briefly, cell lines and primary myeloma cells were seeded in RPMI media containing 2 % human serum and 1 ng/ml IL-6 (ANBL-6 and INA-6 cells), and incubated with drugs as indicated for 3 days in the absence or presence of HCQ, respectively. The viability of the cell lines was unaffected by 10 μM HCQ whereas this dose resulted in approximately 10 % cell death compared to control in some of the primary plasma cell isolates (data not shown). We therefore show data applying 3 μM HCQ with the primary plasma isolates. Cell viability were measured after staining with the apoptotic marker YO-PRO-1 (1 μM, Invitrogen, Carlsbad, CA, USA) and nuclear stain DRAQ5 (2.5 μM, eBioscience, San Diego, CA, USA). Cell stainings were quantified as earlier described applying ScanR automated image acquisition and analysis [18]. INA-6 cells made tolerant to carfilzomib were treated with carfilzomib in the absence or presence of 20 μM HCQ over night before analysis of cell viability as earlier described [18].

CellTiter-Glo (Promega, Madison, WI, USA) was used to determine cell viability in INA-6 cells overexpressing SQSTM1. Cells were seeded in 96-wells plates, treated as indicated, before measurement of cell viability according to the manufacurer's protocol. Luminesence was determined with a Victor 1420 multilabel counter (Perkin Elmer Inc., Waltham, MA, USA).

### Immunoblotting

Cells were washed with ice cold phosphate-buffered saline (PBS) and lysed in 8 M urea, 0.5% Triton X-100 and 0.1 M dithiothreitol. The samples were sonicated to shear DNA before electrophoresis using NuPAGE Bis-Tris gels with MOPS running buffer (Invitrogen, Carlsbad, CA, USA). Gels were blotted onto nitrocellulose membranes, blocked with 5% nonfat dry milk in Tris-buffered saline with 0.1% Tween 20 (TBS-T) and incubated over night with primary antibodies as indicated. Primary antibodies used were; SQSTM1 (cat. no GP62-c, Progen Biotechnik, Heidelberg, Germany), or mouse anti-SQSTM1 antibody from BD Biosciences, catalog No. 610832); anti-GAPDH (cat. no Ab8245, Abcam, Cambridge, UK). Blots were washed in TBS-T before incubation for one hour with HRP conjugated (Dako Cytomation, Glostrup, Denmark) or IRDye conjugated (LI-COR Biosciences, Ltd., Cambridge, UK) secondary antibodies. Bands were detected using SuperSignal West Femto (Thermo Fisher Scientific, Waltham, MA, USA) as luminescence substrate and Odyssey Fc imager (LI-COR Biosciences). Ratios between signal strengths in different bands were calculated using Image Studio software (LI-COR Biosciences).

### *In vitro* proteasome assay

Experiments were done according to the assay-protocol for Proteasome-Glo Chymotrypsin-like Cell-based kit (Promega Corporation, Madison, WI, USA, cat. no G8660), applying purified Human 20S Proteasomes (Boston Biochem, Cambridge, MA, USA, cat. no E-360-050). The experiments were done in 96-well white-wall luminescence plates, using 1 μg/ml proteasomes in 50 μl PBS/well. After adding proteasomes/carfilzomib/HCQ, the plate was incubated at 37°C, 5% CO_2_. After 1 hour incubation, 50 μl Proteasome-Glo reagent was added to wells, after 15 min incubation in RT and luminescence was read on Victor 1420 multilabel counter (PerkinElmer Inc., Waltham, MA, USA).

### Statistical analysis

Statistical analysis was performed using the Prism 4 software. Non-linear regression analysis was employed to fit the dose response curves and to calculate IC50 values. Extra sum-of-squares F test was used to test whether IC50 values differed between groups. For statistical analysis data from at least three independent experiments were used in each case, with two technical repeats for each condition. Statistical significance was tested with two-tailed Student's T-test for comparison of two groups. A two-way between groups analysis of variance (ANOVA) was used to compare multiple groups. Levels of statistical significance (p values) are indicated in figures and Table 1.

## SUPPLEMENTARY FIGURES AND TABLE


