# Supplementary material for: Hydroxychloroquine potentiates carfilzomib toxicity towards myeloma cells
Source: Oncotarget. 2016 Sep 23;7(43):70845–56. doi: 10.18632/oncotarget.12226 (PMC5342593; doi:10.18632/oncotarget.12226)
Supplement: Supplementary file 1 [file oncotarget-07-70845-s001.pdf]

Hydroxychloroquine potentiates carfilzomib toxicity towards myeloma cells

SUPPLEMENTARY FIGURES AND TABLE

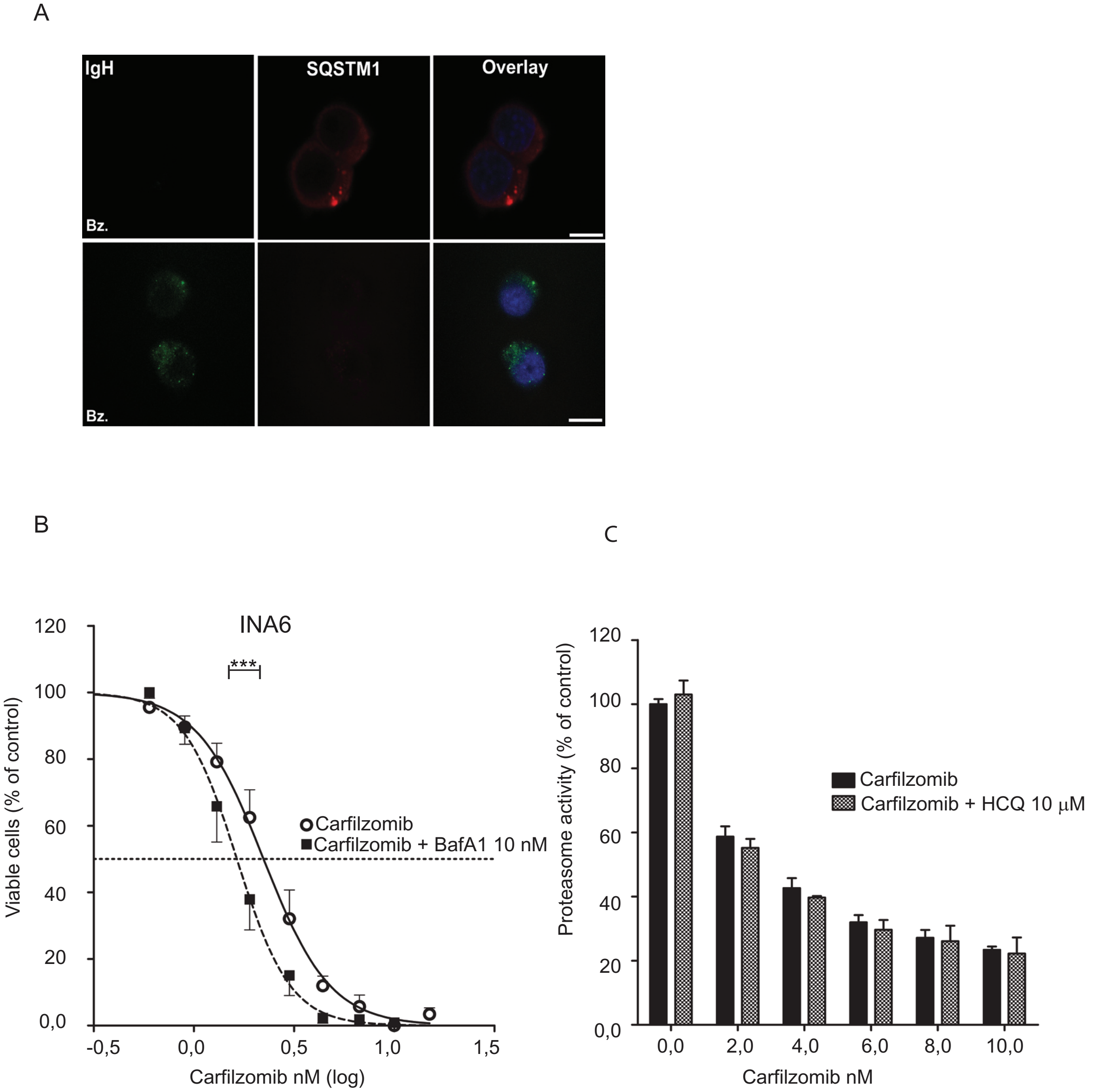

Supplementary Figure S1: A. Specificity control of the antibodies used for IgH and SQSTM1 protein visualization. Bortezomib-treated cells were stained with cocktails of donkey anti-goat Alexa Fluor 555 and chicken anti-rabbit Alexa Fluor 647 omitting the primary anti-IgH staining (upper left panel) or anti-SQSTM1 staining (lower middle panel) to verify the specificities of antibody staining. DNA was visualized using Hoechst 33342 staining. Scale bar is 10 µM. **B.** Bafilomycin A1 potentiates carfilzomib-induced myeloma cell death. INA-6 cells were incubated with indicated concentrations of carfilzomib in the presence (closed squares), or absence (open circles) of 10 nM BafilomycinA1 (BafA1) for 48 hours, before measurement of cell viability were performed as described in Materials and Methods. Results are shown as the mean +-SEM (Standard Error of the Mean) of 3 independent experiments. IC50 was calculated with the use of non-linear regression analysis. Extra sum-of-squares F test was used to test whether IC50 values differed between groups. (***; $p<0.001$). **C.** HCQ does not affect proteasome activity or carfilzomib induced inhibition of proteasome activity. Isolated mammalian proteasomes (1 µg/ml) were incubated with indicated doses of carfilzomib and HCQ for 60 min at room temperature before measurement of proteasome activity as described in Materials and Methods.

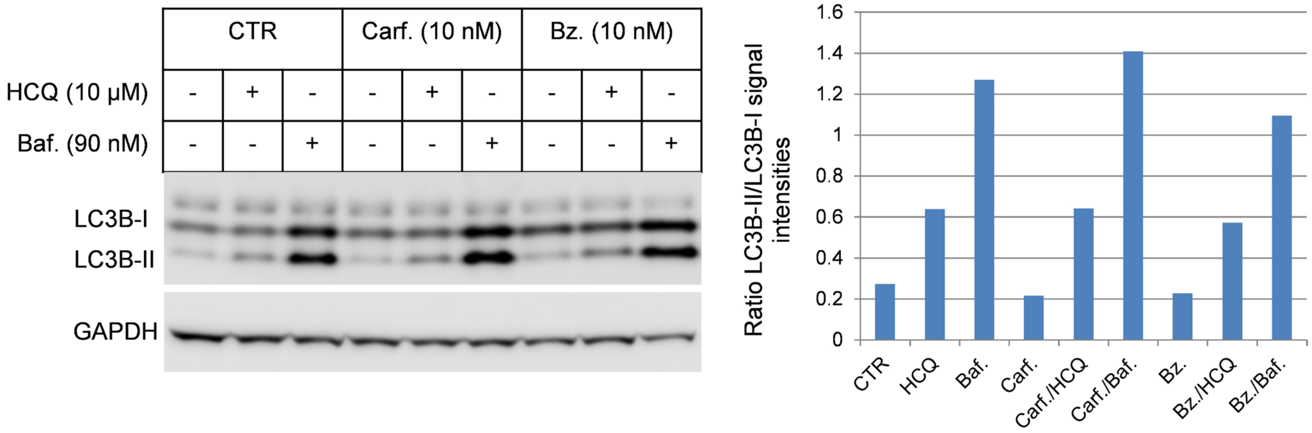

Supplementary Figure S2: ANBL-6 cells were seeded in 2 % HS in RPMI and treated with the indicated doses of HCQ, BafA1, carfilzomib, and bortezomib. After 18-hour incubation, the cells were used for immunoblotting with antibodies targeting LC3B or GAPDH (left hand panel). The LC3B antibody detects both LC3B-I and LC3B-II. The LC3B-II/LC3B-I signal intensity ratios were calculated and are shown in the right hand panel.

Supplementary Table S1: Multiple myeloma patient characteristics

Patient	Age	Sex	HC	LC	Disease stage	TFD	Previous treatment
**MM1**	66	F	IgG	Kappa	D		-
**MM2**	74	F	IgG	Lambda	D		-
**MM3**	45	M	IgG	Kappa	R	3 y	Vel, Cyclo, Dex, HD- Melph
**MM4**	73	M	IgA	Lambda	D		-
**MM5**	77	F	nd		R	2 y	MPT

HC = Heavy Chain, LC = Light Chain, D = Diagnosis, R=Relapse, TFD = Time From Diagnosis, Vel = Velcade, Cyclo = Cyclophosphamide, Dex = Dexamethasone, HD-Melph = High dose Melphalan, MPT = Melphalan, Prednisolone, Thalidomide, nd=not determined.
